# Identification of tuberculosis-associated proteins in whole blood supernatant

**DOI:** 10.1186/1471-2334-11-71

**Published:** 2011-03-22

**Authors:** Takahiro Tanaka, Shinsaku Sakurada, Keiko Kano, Eri Takahashi, Kazuki Yasuda, Hisashi Hirano, Yasushi Kaburagi, Nobuyuki Kobayashi, Nguyen Thi Le Hang, Luu Thi Lien, Ikumi Matsushita, Minako Hijikata, Takafumi Uchida, Naoto Keicho

**Affiliations:** 1 Molecular Enzymology, Graduate School of Agricultural Science, Tohoku University, 1-1 Amamiya-machi, Tsutsumidori, Aoba-ku, Sendai, Miyagi 981-8555, Japan; 2 Department of Respiratory Diseases, Research Institute, National Center for Global Health and Medicine, 1-21-1 Toyama, Shinjuku-ku, Tokyo 162-8655, Japan; 3 Department of Diabetic Complications, Research Institute, National Center for Global Health and Medicine, 1-21-1 Toyama, Shinjuku-ku, Tokyo 162-8655, Japan; 4 Department of Metabolic Disorder, Research Institute, National Center for Global Health and Medicine, 1-21-1 Toyama, Shinjuku-ku, Tokyo 162-8655, Japan; 5 Supramolecular Biology, International Graduate School of Art and Sciences, Yokohama City University, 1-7-29 Suehirocho, Tsurumi-ku, Yokohama, Kanagawa 230-0045, Japan; 6 Department of Respiratory Medicine, National Center for Global Health and Medicine, 1-21-1 Toyama, Shinjuku-ku, Tokyo 162-8655, Japan; 7 National Center for Global Health and Medicine - Bach Mai Hospital (NCGM-BMH) Medical Collaboration Center, 78 Giai Phong St., Hanoi, Vietnam; 8 Hanoi Tuberculosis and Lung Disease Hospital, 44 Thanh Nhan Road, Hanoi, Vietnam

## Abstract

**Background:**

Biological parameters are useful tools for understanding and monitoring complicated disease processes. In this study, we attempted to identify proteins associated with active pulmonary tuberculosis (TB) using a proteomic approach.

**Methods:**

To assess TB-associated changes in the composition of human proteins, whole blood supernatants were collected from patients with active TB and healthy control subjects. Two-dimensional difference gel electrophoresis (2D-DIGE) was performed to analyze proteins with high molecular weights (approximately >20 kDa). Baseline protein levels were initially compared between patients with active TB and control subjects. Possible changes of protein patterns in active TB were also compared *ex vivo *between whole blood samples incubated with *Mycobacterium tuberculosis *(*Mtb*)-specific antigens (stimulated condition) and under unstimulated conditions. Immunoblot and enzyme-linked immunosorbent assays (ELISA) were performed to confirm differences in identified proteins.

**Results:**

Under the baseline condition, we found that the levels of retinol-binding protein 4 (RBP4), fetuin-A (also called α-HS-glycoprotein), and vitamin D-binding protein differed between patients with active TB and control subjects on 2D gels. Immunoblotting results confirmed differential expression of RBP4 and fetuin-A. ELISA results further confirmed significantly lower levels of these two proteins in samples from patients with active TB than in control subjects (*P *< 0.0001). *Mtb*-specific antigen stimulation *ex vivo *altered clusterin expression in whole blood samples collected from patients with active TB.

**Conclusions:**

We identified TB-associated proteins in whole blood supernatants. The dynamics of protein expression during disease progression may improve our understanding of the pathogenesis of TB.

## Background

Tuberculosis (TB) is one of the most important infectious causes of death worldwide [1]. Despite its long historical interaction with humans, our understanding of host response to the TB pathogen remains incomplete. Investigation of the molecular basis of differences in the host immune status and metabolism between patients with active TB and control subjects may provide a clue to understand the disease process, and thus contribute to future strategies for TB prevention and treatment.

Recent advances in comprehensive analytical techniques, such as transcriptomics and proteomics, have enabled us to identify proteins associated with active TB in humans. As a pioneering approach, Jacobsen et al. compared the gene expression profiles of peripheral blood mononuclear cells from patients with TB and *Mtb*-infected healthy donors by microarray analysis [2], and Mistry et al. analyzed gene expression patterns in whole blood in an attempt to find a candidate biomarker for discriminating cured patients from those with a risk of relapse [3].

Agranoff et al. [4] identified amyloid A and transthyretin in human serum as potential indicators for distinguishing patients with TB from those with non-TB inflammatory conditions. They also reported that a combination of four protein markers, including amyloid A and transthyretin, achieved a diagnostic accuracy of up to 78%. Chegou et al. [5] reported that EGF, VEGF, TGF-α, and sCD40L in supernatants obtained from interferon-gamma (IFN-γ)-release assays (IGRAs) are informative markers for differentiating active disease from latent infection. Although the above studies are promising, such comprehensive analytical techniques are still in the developmental stages and further investigations are required before they can be applied clinically.

IGRA detects TB infection by measuring the *Mtb*-specific immune response with high specificity [6]. IFN-γ is released by reactivation of *Mtb*-specific effector memory T cells in whole blood. Despite its advantages, IGRA is not a perfect tool for use in most developing countries. In countries with a high TB burden, patients with active TB, and not those with latent TB infection, need to be immediately identified and treated in order to prevent disease transmission. However, IGRA is not capable of distinguishing active TB from latent infection. Also, cytokine measurements to be performed for IGRA are rather expensive in a resource-limited setting and difficult to distribute. Thus, from a practical as well as a research standpoint, development of new markers for TB is desired.

In the present study, by high-resolution two-dimensional difference gel electrophoresis (2D-DIGE) followed by liquid chromatography-mass spectrometry (LC-MS), we analyzed the expression profiles of high molecular weight proteins (approximately >20 kDa) that have not been studied fully among components of residual whole blood supernatants after performing IGRA.

We used two comparative frameworks. One was the direct comparison of plasma supernatants collected from patients with active TB and healthy control subjects. This comparison aimed to identify proteins that are markedly upregulated or downregulated in the disease state; even if such proteins are not disease specific, they might act as useful markers for monitoring the disease before, during, and after treatment. The other comparative framework was more TB specific since whole blood samples from patients were stimulated with *Mtb-*specific antigens or left unstimulated, and the results were compared.

## Methods

### Patients and control subjects

In this study, whole blood samples collected from Japanese and Vietnamese individuals were used. The study was approved by the ethical review committees of the National Center for Global Health and Medicine (formerly the International Medical Center of Japan), Tokyo, Japan, and the Ministry of Health, Vietnam. Written informed consent was obtained from each participant. Blood samples were collected from patients with active TB immediately before (Vietnamese patient samples) or within 7 days (Japanese patient samples) of treatment initiation. Patients with potential complications attributable to malignancies, autoimmune diseases, or HIV coinfection were excluded from the study.

At the initial screening and confirmation stage, blood samples were collected from 14 Japanese patients with bacteriologically confirmed active pulmonary TB (9 men and 5 women; median age 50 years, range 22-75 years) and 13 age- and gender-matched healthy Japanese patients (8 men and 5 women; median age 48 years, range 24-64 years). We could not completely rule out the possibility of latent TB infection in 2 of the 13 control subjects, according to the results of a commercially available IGRA (QuantiFERON^®^-TB Gold in Tube; Cellestis, Victoria, Australia). However, we analyzed all samples together at the initial stage to identify proteins associated with active TB disease. The tuberculin skin test was not useful for detecting latent TB infection in our study since most individuals in the tested populations had received BCG vaccination after birth. Blood samples from 4 patients with active TB and 4 healthy individuals were chosen for screening by 2D-DIGE and immunoblotting. The stability of proteins measured by the enzyme-linked immunosorbent assay (ELISA) was investigated by comparing a set of plasma samples directly separated from EDTA-containing peripheral blood and another set of plasma supernatants obtained from heparinized blood after 18 h of incubation (under the same conditions as the IGRA negative control).

At the next verification stage, we utilized samples from 25 Vietnamese patients with sputum smear-positive active pulmonary TB (13 men and 12 women; median age 35 years, range 20-55 years) and 50 age- and gender-matched Vietnamese healthy control subjects (26 men and 24 women; median age 36 years, range 21-54 years) of which 25 were IGRA positive and 25 were IGRA negative. None of the IGRA-positive individuals had any signs or symptoms of active TB but at least some were reasonably suspected to have latent TB infections because the prevalence of TB in the population is high. Following IGRA, the remaining unstimulated plasma supernatants were used for ELISA.

### Sample collection and preparation

Whole blood was separately collected in heparin-containing tubes precoated with mitogen as a positive control or cocktails of ESAT-6, CFP-10, and TB7.7 (p4) peptides as *Mtb*-specific antigens (QuantiFERON^®^-TB Gold in Tube; Cellestis); the negative control tubes had no precoat. After 18 h of incubation at 37°C, each sample was centrifuged and the plasma supernatants were harvested and stored at -80°C until use in subsequent assays. For proteomic analysis, four sample sets that were either unstimulated or stimulated with *Mtb*-specific antigens or mitogen from patients with active pulmonary TB and four corresponding sets from control subjects were used to screen for candidate proteins by 2D-DIGE. To increase resolution, 14 human major plasma proteins (albumin, IgG, antitrypsin, IgA, transferrin, haptoglobin, fibrinogen, alpha 2-macroglobulin, alpha 1-acid glycoprotein, IgM, apolipoprotein Al, apolipoprotein All, complement C3, and transthyretin) were removed prior to electrophoresis using a Multiple Affinity Removal LC Column-Human 14 (Agilent Technologies, Santa Clara, CA, USA). The samples were then concentrated by ultrafiltration (Agilent Technologies, Concentrators Spin 5 kDa MWCO, 4 ml) followed by acetone precipitation in preparation for subsequent electrophoresis.

### Quantitative analyses by 2D-DIGE

Protein samples were labeled with Cy3 and Cy5 (DIGE Fluors Minimal Labeling Dyes; GE Healthcare, Buckinghamshire, UK) according to the manufacturer's instructions. The samples (50 μg of total protein per gel) were applied to Immobiline DryStrips (18 cm long, pH 4-7 linear; Amersham Biosciences, Pittsburgh, PA, USA), and isoelectric focusing (IEF) was performed using an Ettan IPGphor IEF system (Amersham Biosciences) according to the manufacturer's instructions. Next, SDS-PAGE was performed using a 10-18% linear gradient gel from DRC Co., Ltd. (Tokyo, Japan). The fluorescence intensity of each protein spot was digitally recorded using a Molecular Imager FX system (Bio-Rad Laboratories, Hercules, CA, USA) with Quantity One software (Bio-Rad Laboratories), and differential protein expression was quantitatively analyzed using the PDQuest software (Bio-Rad Laboratories). The same gel included a reference sample that had been labeled with Cy2 and was used for spot matching, image analysis, and volume normalization. Initially, all spots were roughly matched using an automated tool in the PDQuest software suite. This estimate was followed by a more detailed manual curation to correct any inappropriately matched pairs of protein spots.

### Sample preparation for mass spectrometry

A mixture of all samples (400 μg of total protein per gel) was subjected to 2D-DIGE under the same conditions as described above to isolate selected spots. To visualize individual protein spots, the gels were stained with SYPRO Ruby protein gel stain (Molecular Probes, Eugene, OR, USA) for 3 h. The fluorescence intensity of each protein spot was digitally measured using the Molecular Imager FX system with Quantity One software. Mass spectrometric analysis was performed according to the method reported by Toda *et al. *[7], with slight modification. Briefly, each protein spot on SYPRO Ruby stained gels was picked using a spot picker (Amersham Biosciences). In-gel digestion of proteins was performed according to the method reported by Saeki *et al. *[8].

### Mass spectrometric analysis

An ESI ion-trap mass spectrometer (LCQ Deca XP Plus, Thermo Electron) was used for peptide detection. Mass spectrometric analysis was performed as described previously [8]. Protein identification was performed using the Mascot server (Matrix Science, Boston, MA, USA) and Protein Prospector (UCSF Mass Spectrometry Facility, San Francisco, CA, USA). We selected the SWISS-PROT *Homo sapiens *database and used the following parameters: peptide tolerance 1.0 Da and one missed cleavage. Carbamidomethyl modification of cysteine, acetylation of the NH2-terminal ends of lysine, and phosphorylation of serine, threonine, or tyrosine were considered in this analysis.

### Immunoblotting

Immunoblotting to detect the proteins identified as described above was performed using anti-human retinol-binding protein 4 (RBP4) rabbit polyclonal IgG (A-0040; Dako; Glostrup, Denmark), anti-human fetuin-A (AHSG) goat polyclonal IgG (G-20; Santa Cruz Biotechnology; Santa Cruz, CA, USA), anti-human vitamin D-binding protein (VDBP) (Gc-Globulin) rabbit polyclonal IgG (Dako), anti-human clusterin-α mouse monoclonal IgG1 (B-5; Santa Cruz Biotechnology), or anti-human clusterin-β rabbit polyclonal IgG (N-18; Santa Cruz Biotechnology).

Total protein concentrations were determined using the Bio-Rad protein assay kit (Bio-Rad Laboratories). To detect clusterin-α and -β, mixed protein samples (20 μg) were applied to 2D PAGE with 1D IEF using the Immobiline DryStrip (pH 3-5.6 nonlinear). Proteins were then transferred to PVDF membranes. The membranes were probed with polyclonal antibodies, anti-clusterin-α, and anti-clusterin-β. To detect other proteins, each sample (10 μg) was subjected to conventional SDS-PAGE. Membranes were probed with anti-VDBP, anti-fetuin-A, or anti-RBP4 polyclonal antibodies. Anti-mouse and anti-rabbit (GE Healthcare) as well as anti-goat (Santa Cruz Biotechnology) HRP-conjugated secondary antibodies were prepared. Protein bands were detected using the ECL plus detection reagent (GE Healthcare). Band intensities were calculated using the Quantity One software.

### ELISA

A competitive ELISA for quantitative determination of RBP4 in human plasma was performed according to the manufacturer's instructions (AdipoGen Inc.; Seoul, Korea). The detection limit was 1 ng/ml. An AHSG ELISA kit was used to detect fetuin-A in plasma (BioVender Laboratory Medicine Inc.; Modrice, Czech Republic). The detection limit was 0.35 ng/ml. A Quantikine^® ^Human Vitamin D-Binding Protein Immunoassay kit was used to detect VDBP in plasma (R&D Systems, Inc.; Minneapolis, MN, USA). The mean minimum detectable VDBP level was 0.65 ng/ml. Distribution of levels was represented using the median and interquartile range (IQR).

### Statistical analysis

Proteins showing differential expression between two conditions were first determined with *P *values using the Student's *t*-test preinstalled in the PDQuest software suite. To select candidate proteins with expression levels that differed between unstimulated samples from patients with active TB and healthy control subjects, a significance level of *P *< 0.05 was selected. To select candidate proteins showing differential expression in *Mtb-*specific antigen-stimulated and unstimulated plasma samples, a less stringent cut-off value of *P *< 0.10 was applied. Assuming an alpha error of 0.1 and a standardized effect size of 2.0, the power to detect a difference was calculated as 0.8 given our sample size. When a normal distribution of measurements was not predicted, the Wilcoxon rank sum test (Mann-Whitney U test) was applied for confirmation using the JMP software (version 7.0.1; SAS Institute, Cary, NC, USA).

## Results

### Quantitative analyses by 2D-DIGE

**Figure 1:**
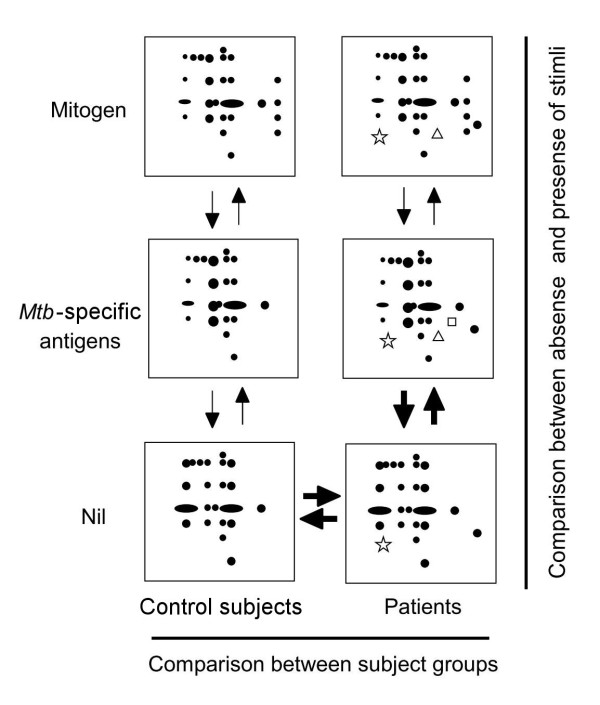
**Schematic representation of protein expression profiles**. Comparison between subjects, in particular, spot patterns showing differences in plasma proteomes between patients and control subjects. Comparison between the presence and absence of stimuli, especially *ex vivo *spot patterns of *Mtb-*specific antigen-stimulated plasma samples and unstimulated samples from patients. Two major comparative frameworks in our study are shown (bold arrows).

In a preliminary experiment, we used an immobilized linear pH gel strip with a broad pH range (pH 3-10 linear) for 1D IEF. Although more than 500 protein spots were visualized in fresh plasma with SYPRO Ruby staining, the number of spots after incubation of whole blood with stimuli decreased, and detectable spots were primarily located in the pH range 4-7 (data not shown). Therefore, we performed subsequent analyses using an immobilized linear pH gel strip with a narrower range (pH 4-7 linear) to obtain a finer resolution. We used two comparative frameworks in our analyses, and the corresponding spot patterns are schematically depicted in Figure 1.

**Figure 2:**
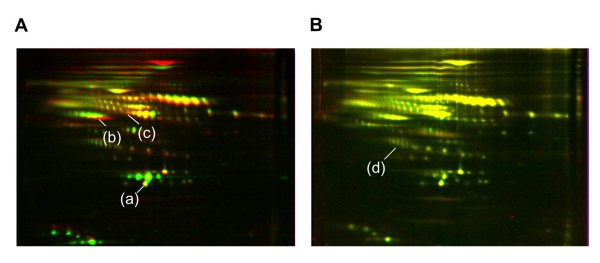
**2D-DIGE-based protein expression analysis**. Pseudo-colored images were generated using the Multi-Channel Viewer function of the PDQuest software suite. Four sample sets that were either unstimulated or stimulated with the *Mtb-*specific antigen or mitogen from patients with active pulmonary TB and four corresponding sets from control subjects were used to screen for candidate proteins by 2D-DIGE. A: Analysis based on differences in levels in samples from patients and control subjects. Red indicates proteins whose levels increased and green indicates proteins whose levels decreased in unstimulated supernatants from patients with active TB compared with control subjects. Spot (a): HT6102, Spot (b): HT2406, and Spot (c): HT5401 correspond to the PDQuest identification numbers shown in Table 2. B: Analysis based on differences in levels after stimulation. Red indicates proteins whose levels increased in whole blood supernatants from patients with active TB after incubation with *Mtb-*specific antigens. Spots (d): T3107, T4203, and T4208, correspond to Table 2.

Differential gel images were acquired and displayed using the PDQuest 2D gel analysis software (Figure 2A, B). In our comparison of the protein expression profiles of patients with active TB and control subjects, red indicates proteins increased in the supernatants collected from the patients and green indicates proteins decreased in the patients compared with the control subjects. Yellow indicates no significant differences (Figure 2A). In 2D gel profiles comparing the antigen-stimulated and unstimulated samples collected from patients with active TB, red indicates proteins increased in the supernatants after *Mtb*-specific antigen stimulation, and green indicates proteins decreased after stimulation. Yellow indicates no significant changes (Figure 2B). From 367 spots compared between patients with active TB and control subjects, and 293 spots generated with samples collected from patients with active TB that were either stimulated with *Mtb*-specific antigens or left unstimulated, we selected several candidates for subsequent mass spectrometric analysis (Table 1) according to the criteria described in the Materials and Methods section.

**Table 1:** The number of spots that may show differential expression.

A: Comparison between patients with active TB and control subjects				
		***P *< 0.02**	**0.02≤*P < 0.05***	**0.05≤*P *< 0.10**

Patients versus control subjects		18	12	24

**B: Comparison between stimulated and unstimulated conditions**				

		**P < 0.02**	**0.02≤P < 0.05**	**0.05≤P < 0.10**

Patients	*Mtb *antigens versus no stimuli	0	2	2
	
	Mitogen versus no stimuli	3	5	11

Control subjects	*Mtb *antigens versus no stimuli	0	1	13
	
	Mitogen versus no stimuli	2	83	8

### Mass spectrometric analysis

**Figure 3:**
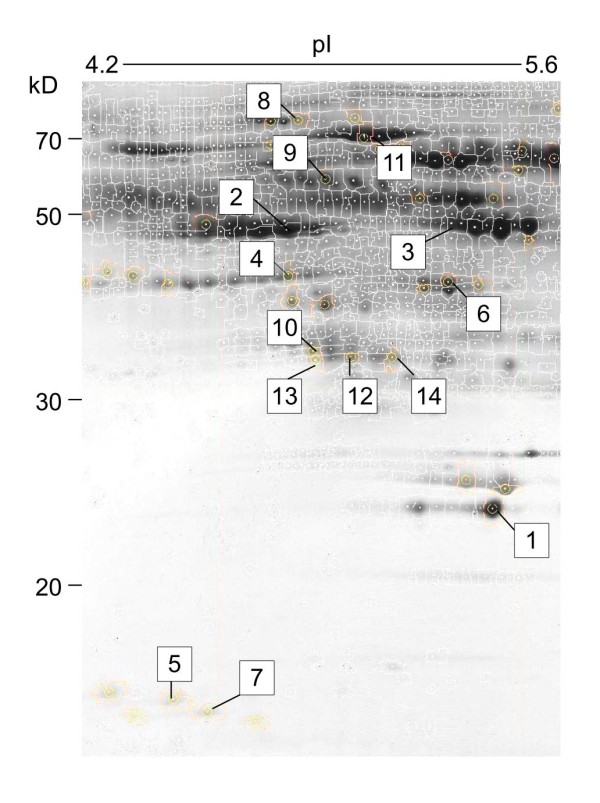
**Representative 2D gel image obtained from pooled protein samples**. Differentially expressed proteins successfully identified by LC-MS were represented on the gel using the PDQuest software suite. See Table 2 for additional information. Numbers in the figure correspond to serial numbers in Table 2.

Following the above criteria for selecting candidates of differentially expressed proteins between two conditions, a total of 41 spots were isolated from the corresponding 2D gels on the basis that they showed sufficiently strong signals. Trypsin digestion of each isolated spot was followed by LC-MS analysis. The proteins corresponding to 14 of these spots were successfully identified (Figure 3).

Of the 14 proteins in Table 2, 7 (serial numbers 1 to 7) were obtained as a result of comparisons between patients with active TB and control subjects; number 1 (spot HT6102) was identified as RBP4, number 2 (HT2406) as fetuin-A, and number 3 (HT5401) as VDBP. Four (numbers 8 to 11) were obtained as a result of comparisons between nonspecific mitogen-stimulated and unstimulated samples collected from patients with active TB (not analyzed in this study). The last 3 proteins (numbers 12 to 14) were obtained as a result of comparisons between *Mtb*-specific antigen-stimulated and unstimulated samples collected from patients with active TB; numbers 12 to 14 (T4203, T3107, and T4208) were all identified as clusterin. In Table 2, *P *values indicating a significant difference between the means of the two conditions examined, the SWISS-PROT accession numbers of the identified proteins as well as their molecular weights and theoretical pI values are indicated. We also used the *Homo sapiens *database of expressed sequence tags (ESTs) to identify clusterin in spot T4208.

**Table 2:** Characteristics of proteins identified in this study.

A: Comparison between patients with active TB and control subjects									
		**2D-DIGE**			**LC-MS/MS**				

**Condition**	**Serial Number**	**PDQ SSP#^a^**	***P *value**	**+/-^b^**	**Swiss-Plot**	**Mascot search score^e^**	**Protein name**	**Da**	**pI**

Patients versus control subjects	1	HT6102	0.0064	-	RET4_HUMAN	72	Retinol binding protein 4	23010	5.76
	2	HT2406	0.0097	-	FETUA_HUMAN	75	α-2-HS-glycoprotein	39325	5.43
	3	HT5401	0.0331	+	VTDB_HUMAN	98	Vitamin D binding protein	52964	5.40
	4	HT2303	0.0419	+	CO4A_HUMAN	86	Complement C4A	192771	6.66
	5	HT1012	0.0271	-	APOC3_HUMAN	105	Apolipoprotein C-III	10852	5.23
	6	HT5303	< 0.001	-	APOA4_HUMAN	190	Apolipoprotein A-IV	45399	5.28
	7	HT1016	0.0024	-	APOC2_HUMAN	61	Apolipoprotein C-II	11284	4.72

**B: Comparison between stimulated and unstimulated conditions in active TB**									

		**2D-DIGE**			**LC-MS/MS**				

**Condition**	**Serial Number**	**PDQ SSP#**	***P *value**	**+/-^c^**	**Swiss-Plot**	**Mascot search score**	**Protein name**	**Da**	**pI**

Mitogen (versus no stimuli)	8	T3601	0.0917	-	C1S_HUMAN	169	Complement-C1S	76684	4.86
	9	T3403	0.0156	+	KNG1_HUMAN	139	Kininogen-1	71957	6.34
	10	T3105	0.0866	-	ZA2G_HUMAN	45	Zinc-α-2-glycoprotein	33872	5.57
	11	T4512	0.0061	-	A1BG_HUMAN	76	α-1B-glycoprotein	54273	5.58

*Mtb *antigens (versus no stimuli)	12	T4203	0.0640^d^	+	CLUS_HUMAN	47	Clusterin	52495	5.89
	13	T3107	0.0687^d^	+	CLUS_HUMAN	50	Clusterin	52495	5.89
	14	T4208	0.0732^d^	+	EST	--	Clusterin	52495	5.89

^a^PDQ SSP# is a PDQuest software standard spot number indicating the unique location of each spot automatically assigned on a 2D gel and is essential for comparing the same spots on different gels.

^b^The average density of a spot from 2D-DIGE is higher (+) or lower (-) in patients with active TB than in control subjects.

^c^The average density of the spot from 2D-DIGE is higher (+) or lower (-)under the stimulated condition than that under the unstimulated condition.

^d^The average density of the 3 spots, T4203, T3107, and T4208, which correspond to a subset of clusterin, was significantly higher in *Mtb-*specific antigen-stimulated than in unstimulated samples (*P *= 0.0014).

^e^The Mascot search score indicates the degree of compatibility between mass spectra generated by the sample and amino acid sequences within the protein of interest.

Mascot search scores (indices of protein matches) were 47, 50, 98, 75, and 72 for spots T4203 (clusterin), T3107(clusterin), HT5401 (VDBP), HT2406 (fetuin-A), and HT6102 (RBP4), respectively, (Table 2), suggesting that identification of these proteins using peak lists of MS/MS spectra obtained from the LC-MS/MS system are fairly reliable since all these scores were significant above the 5% confidence threshold and no other proteins with comparable scores were detected for each gel spot (See Additional file 1: for supporting information). These proteins were interesting because of their potential biological significance, and we therefore analyzed them further.

### Confirmation of differentially expressed proteins by immunoblotting

**Figure 4:**
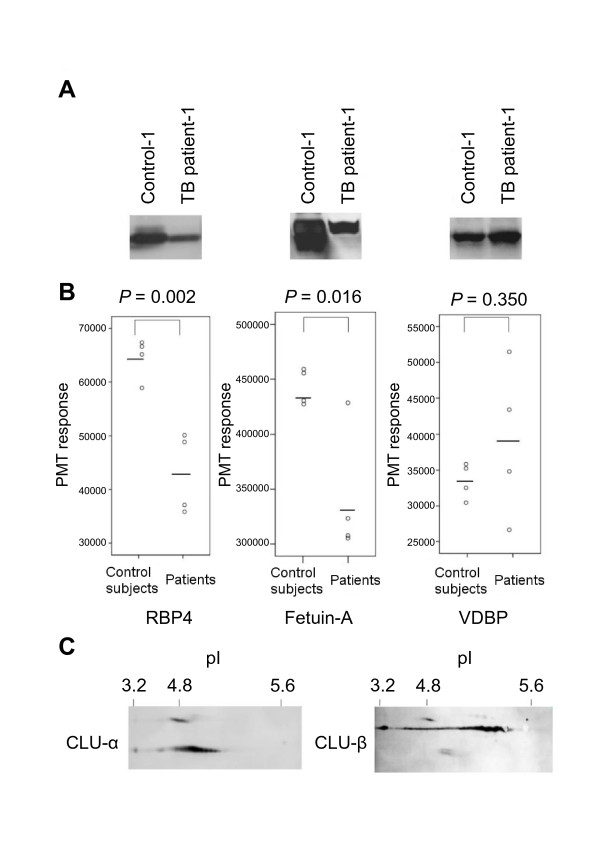
**Comparison of candidate differential proteins using immunoblotting**. Samples were first treated to remove 14 major proteins and then analyzed by immunoblotting to facilitate comparison of RBP4, fetuin-A, and VDBP levels. (A) Representative immunoblot of samples from patient with active TB (Patient-1) versus a control subject (Control-1). (B) Band densities were quantified in patients (n = 4) and control subjects (n = 4). The PMT response suggested band densities. (C) Two-dimensional immunoblot of pooled samples with detection of clusterin-α and -β. CLU is clusterin.

Immunoblot analysis was used to confirm differential expression of three proteins identified in patients with active TB compared with control subjects (Figure 4A). We measured band densities using the same samples prepared for protein confirmation (Figure 4B). The band density of RBP4 in patients with active TB (64,283 arbitrary units ± 3,861) was lower than that in control subjects (445,894 ± 16,590), and fetuin-A expression in the patients was also lower (42,710 ± 7,580) than that in control subjects (343,617 ± 58,923). These results are consistent with those of 2D gel analysis. Moreover, the band density of VDBP tended to be higher in samples from patients with active TB than from control subjects, which is similar to that observed above; however, the protein levels were widely distributed and the differences in these levels did not reach significance in the control subjects compared with patients with active TB (33,251 ± 2,572 versus 38,971 ± 11,001). Because the three clusterin spots altered after *Mtb*-specific antigen stimulation were not clearly distinguished by immunoblotting, we did not attempt any further demonstration of changes in these signals in our study. Instead, pooled samples were run on a 2D gel and followed by immunoblotting with anti-clusterin-α and anti-clusterin-β antibodies (because clusterin consists of clusterin-α and -β subunits) (Figure 4C). Based on immunoreactivity and pI values, the spots detected were confirmed to be clusterin-α. More specifically, the three spots comprised a subset of possible modified forms of clusterin-α that may be detected.

### Detection of differentially expressed proteins by ELISA

**Figure 5:**
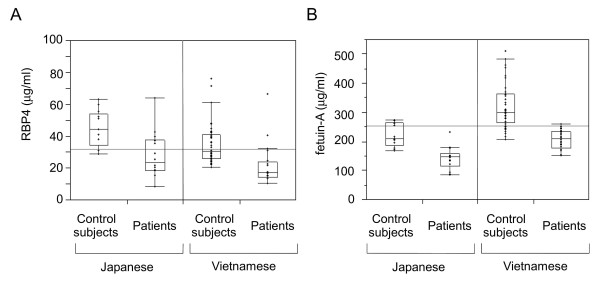
**Comparison of RBP4, fetuin-A, and VDBP levels by ELISA**. Samples were analyzed by ELISA to compare RBP4 and fetuin-A levels in14 Japanese patients with active TB and 13 age-, gender-, and ethnicity-matched control subjects, as well as 25 Vietnamese patients with active TB, and 50 age-, gender-, and ethnicity-matched control subjects. *P *values showing results of comparisons are described in the text. (A) RBP4 levels in patients versus control subjects. (B) Fetuin-A levels in patients versus control subjects.

Because RBP4 and fetuin-A levels determined by immunoblotting were significantly different between samples from patients with active TB and control subjects, we performed further quantitative ELISA to extend the measurements to plasma samples from 14 Japanese patients with active TB and 13 age-, gender-, and ethnicity-matched control subjects. Plasma RBP4 levels in patients with active TB (median = 23.6 μg/ml; IQR = 18.4-37.9) were significantly lower than those from control subjects (median = 44.6 μg/ml; IQR = 34.6-53.8; *P *= 0.0033; Figure 5A). Plasma fetuin-A levels in patients (median = 147.9 μg/ml; IQR = 115.8-159.6) were also significantly lower than those in control subjects (median = 211.0 μg/ml; IQR = 186.7-264.6; *P *= 0.0002; Figure 5B). No significant difference were observed in plasma VDBP levels between patients (median = 110.0 μg/ml; IQR = 85.2-151.3) and control subjects (median = 105.0 μg/ml; IQR = 88.1-215.6; *P *= 0.5441; Figure not shown).

We simultaneously compared the protein levels in plasma immediately separated from EDTA-containing blood with those in plasma supernatants obtained from heparinized blood as a negative control for IGRA after 18 h of incubation without stimulants. We found that the differences between the two types of plasma samples were small (coefficient of variance (CV) = 10.5% for RBP4; CV = 5.0% for fetuin-A; CV = 6.6% for VDBP) and was in a range of variation generally accepted in ELISA (CV < 15%), indicating that the measurements obtained under the latter condition can be substituted for those obtained under the former condition. Indeed, plasma RBP4 and fetuin-A levels in samples from Japanese patients with active TB were significantly lower than those from control subjects, irrespective of plasma conditions (data not shown).

We further attempted to verify the differences observed with samples from a different ethnic and regional population, i.e., samples collected from Vietnamese patients. The two proteins identified above were measured in plasma supernatants from Vietnamese patients with active TB and age-, gender-, and ethnicity-matched control subjects. The samples from these Vietnamese patients were obtained from a negative control of IGRA after incubation without stimulants. RBP4 levels in patients with active TB (median = 17.5 μg/ml; IQR = 14.4-23.9) were significantly lower than those in control subjects (median = 30.5 μg/ml; IQR = 25.9-40.8; *P *< 0.0001; Figure 5A). Fetuin-A levels in patients with active TB (median = 210.7 μg/ml; IQR = 178.1-235.7) were also significantly lower than those in control subjects (median = 299.4 μg/ml; IQR = 265.1-363.2; *P *< 0.0001; Figure 5B). Moreover, both protein levels were not significantly different between IGRA-negative and IGRA-positive subgroups of the control subjects (data not shown).

## Discussion

In this study, we identified TB-associated proteins from whole blood supernatants. After the removal of 14 major plasma proteins, RBP4, fetuin-A, and VDBP were initially identified as plasma proteins from unstimulated samples for which the baseline levels differed between the patients and control subjects. Immunoblotting results confirmed the differential expression of RBP4 and fetuin-A between the two groups. Although VDBP has previously been identified as a biomarker for mycobacterial infections in cattle [9], the level of this protein did not differ significantly in our study because of large individual variations. The changes in VDBP levels may not have been accurately immunologically assayed in our study.

Clusterin is a secreted glycoprotein involved in apoptosis, inflammation, and tissue injury. It was differentially expressed in patients with active TB after stimulation and the intensities of the three spots corresponding to clusterin-α were elevated in whole blood supernatant samples after incubation with *Mtb*-specific antigens. These spots appear to have shifted in both the dimensions on the gel, which suggests small changes in their molecular weights and IEPs. It is conceivable that post-translational modifications, such as degradation and/or deglycosylation, occur via an enzymatic reaction that accompanies immune cell activation. However, we have not demonstrated that this response is observed only when *Mtb*-specific antigens are co-incubated. To determine whether clusterin has a role as a marker of TB or indicates more general response to antigen stimulation, we are currently attempting to find clear and simple methods in detecting these alterations for mass screening.

Subsequent ELISA results for samples from Japanese and Vietnamese subjects confirmed that both plasma RBP4 and fetuin-A levels were significantly lower in samples collected from patients with active TB than in control subjects, indicating that our findings are reproducible in studies using well-matched control subjects. However, as shown in Figure 5, the average plasma levels of these proteins differed between Japanese and Vietnamese control subjects. This suggests that unknown factors may systemically influence tested populations or the measurement of these markers. Because this variance is crucial in a clinical setting, further basic as well as clinical investigations are necessary to accurately assess these markers.

No significant differences were observed in RBP4 and fetuin-A levels in samples from IGRA-positive and -negative control subjects. This suggests that these proteins levels are not affected by latent TB infection, but that they presumably change during disease progression via an unknown mechanism.

Intriguingly, the literature supports the idea that RBP4 and fetuin-A are functionally significant since they may be involved in macrophage activation [10-12]. Retinoic acid has been shown to stimulate and induce monocyte differentiation, leading to inhibition of *Mtb *multiplication in human macrophages [13]. RBP4 is the specific carrier protein for retinol (vitamin A) and has recently been described as an adipokine that contributes to insulin resistance [13]. This protein is believed to modulate pathophysiological processes during bacterial infection. Fetuin-A was originally identified as a fetal protein and has been shown to affect the development of many mammalian tissues. Moreover, the results of *in situ *mRNA hybridization and immunocytochemical studies in adult sheep have revealed that the main sites of fetuin-A expression are hepatocytes and monocytes or macrophages in the spleen and bone marrow [14]. Fetuin-A is known to modulate various immune and metabolic responses. Previous reports have shown that fetuin-A deactivates macrophages, acts as an opsonin for cationic-deactivating molecules including spermine [15], reduces TNF-α production and inflammatory responses [16], and enhances phagocytosis of apoptotic cells and macropinocytosis by human macrophages [17]. On the other hand, this protein is known to be a potent inhibitor of systemic calcification [18] and is associated with the incidence of diabetes mellitus [19].

Our study is the first to highlight the relationship between these two markers and TB, even though these marker levels may be affected by endogenous or exogenous factors and are presumably nonspecific to TB given their relative abundance in plasma and the broad spectrum of functional significance proposed in the above references.

Nevertheless, performing a prospective cohort study may help clarify the role of these proteins in TB. If within-individual variation in baseline levels is relatively small, it can be used to monitor the course of disease before, during, and after treatment. Further clinical studies on various conditions may better characterize these proteins. Single use of these markers or their combined use with other promising biomarkers may be a useful tool to aid the development of new effective therapies and vaccines.

## Conclusions

We identified three TB-associated proteins, RBP4, fetuin-A, and clusterin, in whole blood supernatants using a proteomic approach. We subsequently showed that both plasma RBP4 and fetuin-A levels are significantly and reproducibly lower in patients with active TB than in control subjects. These findings may help us understand and monitor the disease process in TB.

## Abbreviations

TB: Tuberculosis; *Mtb*: *Mycobacterium tuberculosis*; IFN-γ: interferon-gamma; IRGA: interferon-gamma-release assay; 2D-DIGE: two-dimensional difference gel electrophoresis; LC-MS: liquid chromatography-mass spectrometry; ELISA: enzyme-linked immunosolvent assay; IEF: isoelectric focusing; RBP-4: retinol binding protein-4; VDBP: vitamin D binding protein; IQR: interquartile range; ESTs: expressed sequence tags;

## Competing interests

The authors declare that they have no competing interests.

## Authors' contributions

TT carried out the plasma proteome studies, participated in 2D-DIGE studies, a part of immunoassays and drafted the manuscript. SS conceived of the study, and participated in the study planning and coordination and helped to draft the manuscript. KK carried out the LC-MS/MS analysis. ET, KY and HH helped to design the study. YK participated in the study design and overall supervision. NK, NTLH and LTL participated in management and analysis of data. IM and MH participated in the acquisition of data. TU helped to draft the manuscript. NK participated in the design of the study, performed statistical analysis and have given final approval of the version to be published. All authors read and approved the final manuscript.

## Pre-publication history

The pre-publication history for this paper can be accessed here:

http://www.biomedcentral.com/1471-2334/11/71/prepub

## Supplementary Material

Additional file 1 **Figure S1 - Mascot search results--Information about the identified proteins obtained using the Mascot server**. (A) Mascot search result for T2116 (clusterin) (B) Mascot Search Result for T2103 (clusterin) EST (C) Mascot search result for T1486 (clusterin) (D) Mascot search result for HT2482 (RET4 = RBP4) (E) Mascot search result for HT1248 (fetuin-A) (F) Mascot search result for HT1240 (VDBP). Click here for file
