# Supplementary material for: Identification of tuberculosis-associated proteins in whole blood supernatant
Source: BMC Infect Dis. 2011 Mar 22;11:71. doi: 10.1186/1471-2334-11-71 (PMC3072329; doi:10.1186/1471-2334-11-71)

(A) Mascot Search Result T2116 (clusterin)

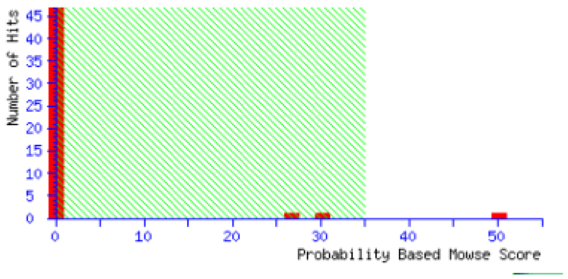

1. sp|P10909|CLUS_HUMAN **Mass:** 53031 **Score:** 50 **Queries matched:** 2 **emPAI:** 0.07
Clusterin OS=Homo sapiens GN=CLU PE=1 SV=1
☐ Check to include this hit in error tolerant search or archive report

	Query	Observed	Mr(expt)	Mr(calc)	Delta	Miss	Score	Expect	Rank	Peptide
☑	218	485.9755	969.9364	969.4477	0.4887	0	50	0.0021	1	**K.FMETVAEK.A + Oxidation (M)**
	293	645.1605	1288.3064	1287.6306	0.6758	0	13	8.9	2	R.ELDESLQVAER.L

Fixed modifications: Carbamidomethyl (C)
Variable modifications: Oxidation (M)
Cleavage by Trypsin: cuts C-term side of KR unless next residue is P
Sequence Coverage: **4%**

Matched peptides shown in **Bold Red**

```
  1 MMKTLLLFVG LLLTWESGQV LGDQTVSDNE LQEMSNQGSK YVNKEIQNAV
 51 NGVKQIKTLI EKTNEERKTL LSNLEEAKKK KEDALNETRE SETKLKELPG
101 VCNETMMALW EECKPCLKQT CMKFYARVCR SGSGLVGRQL EEFLNQSSPF
151 YFWMNGDRID SLLENDRQQT HMLDVMQDHF SRASSIIDEL FQDRFFTREP
201 QDTYHYLPFS LPHRRPHFFF PKSRIVRSLM PFSPYEPLNF HAMFQPFLEM
251 IHEAQQAMDI HFHSPAFQHP PTEFIREGDD DRTVCREIRH NSTGCLRMKD
301 QCDKCREILS VDCSTNNPSQ AKLRRELDES LQVAERLTRK YNELLKSYQW
351 KMLNTSSLLE QLNEQFNWVS RLANLTQGED QYYLRVTTVA SHTSDSDVPS
401 GVTEVVVKLF DSDPITVTVP VEVSRKNPKF METVAEKALQ EYRKKHREE
```

Start - End	Observed	Mr(expt)	Mr(calc)	Delta	Miss	Sequence
326 - 336	645.1605	1288.3064	1287.6306	0.6758	0	**R.ELDESLQVAER.L** (Ions score 13)
430 - 437	485.9755	969.9364	969.4477	0.4887	0	**K.FMETVAEK.A** Oxidation (M) (Ions score 50)

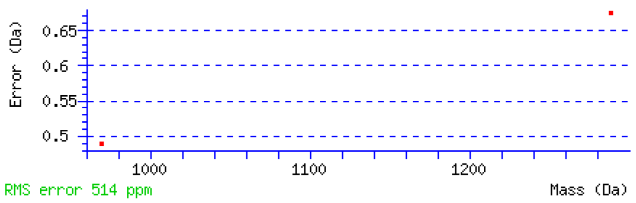

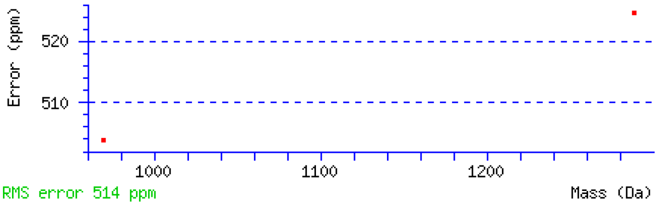

(B) Mascot Search Result T2103 (clusterin) EST

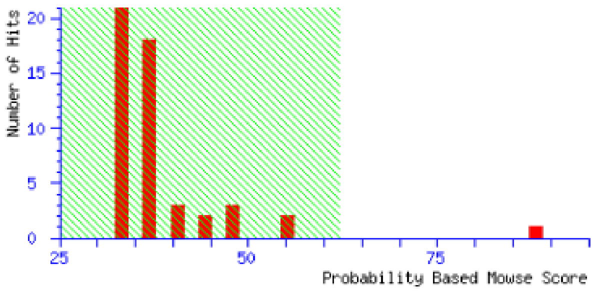

1. gi|4108608 **Mass:** 14825 **Score:** 88 **Queries matched:** 2 **emPAI:** 0.26 **Frame:** 4
qx15a11.x1 NCI_CGAP_Lym12 Homo sapiens cDNA clone IMAGE:2001404 3' similar to gb:X14723 CLUSTERIN PRECURSOI
Check to include this hit in error tolerant search or archive report

Query	Observed	Mr(expt)	Mr(calc)	Delta	Miss	Score	Expect	Rank	Peptide
330	810.1640	1618.3134	1617.8098	0.5037	0	65	0.017	1	-.TSDSDVPSGVTEVVVK.L
375	937.2940	1872.5734	1872.9833	-0.4099	0	23	2.4e+002	8	K.LFDSDPITVTVPVEVSR.K

Proteins matching the same set of peptides:
gi|4108895 **Mass:** 15130 **Score:** 88 **Queries matched:** 2 **Frame:** 6
qx15e07.x1 NCI_CGAP_Lym12 Homo sapiens cDNA clone IMAGE:2001444 3' similar to gb:X14723 CLUSTERIN PRECURSOI
gi|12238427 **Mass:** 11699 **Score:** 88 **Queries matched:** 2 **Frame:** 4
IL5-EN0086-281100-291-e08 EN0086 Homo sapiens cDNA, mRNA sequence
gi|12464764 **Mass:** 10893 **Score:** 88 **Queries matched:** 2 **Frame:** 5
IL5-GN0240-201200-348-d11 GN0240 Homo sapiens cDNA, mRNA sequence

Nominal mass (M_r): **14825**; Calculated pI value: **5.93**
NCBI BLAST search of gi|4108608 against nr
Unformatted sequence string for pasting into other applications

Taxonomy: Homo sapiens

Cleavage by Trypsin: cuts C-term side of KR unless next residue is P
Sequence Coverage: **24%**

Matched peptides shown in **Bold Red**

```
  1 TSDSDVPSGV TEVVVKLFDS DPITVTVPVE VSRKNPKFME TVAEKALQEY
 51 RKKHREE_DV DVAFAPTGAS ESSSPQDELQ PPRESSARHQ VTRPQPPGPQ
101 LRPASPRSGS CTLTLDSAAH GKNRIAPACN _FNKTVL_AE
```

Start - End	Observed	Mr(expt)	Mr(calc)	Delta	Miss	Sequence	
1 - 16	810.1640	1618.3134	1617.8098	0.5037	0	-.TSDSDVPSGVTEVVVK.L	(Ions score 65)
17 - 33	937.2940	1872.5734	1872.9833	-0.4099	0	K.LFDSDPITVTVPVEVSR.K	(Ions score 23)

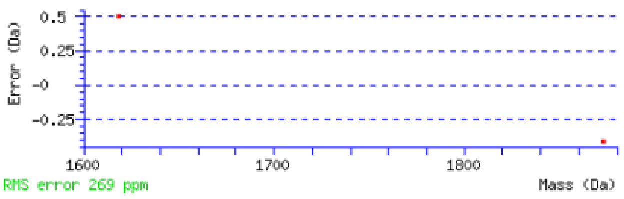

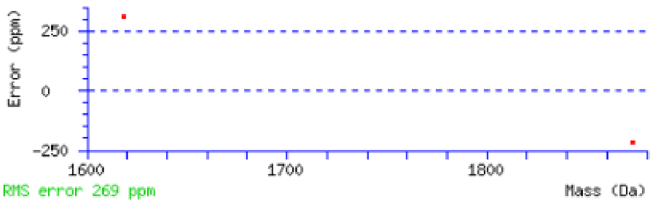

(C) Mascot Search Result T1486 (clusterin)

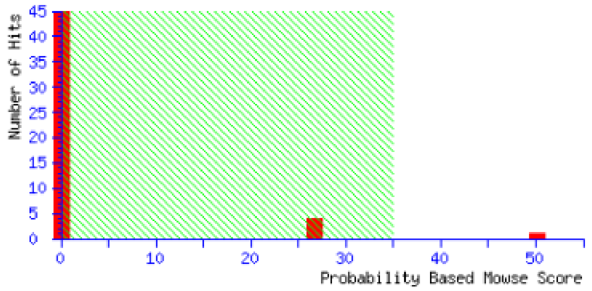

1. sp|P10909|CLUS_HUMAN **Mass:** 53031 **Score:** 50 **Queries matched:** 6 **emPAI:** 0.15
Clusterin OS=Homo sapiens GN=CLU PE=1 SV=1
☐ Check to include this hit in error tolerant search or archive report

	Query	Observed	Mr(expt)	Mr(calc)	Delta	Miss	Score	Expect	Rank	Peptide
☑	144	779.2860	778.2787	778.3973	-0.1186	0	17	4.6	1	K.ALQEYR.K
☑	189	970.3040	969.2967	969.4477	-0.1510	0	24	0.82	1	K.FMETVAEK.A + Oxidation (M)
☑	213	559.3435	1116.6724	1116.6026	0.0698	0	36	0.051	1	K.TLLSNLEEAK.K
☑	242	644.8985	1287.7824	1287.6306	0.1518	0	28	0.31	1	R.ELDESLQVAER.L
☑	383	881.9635	1761.9124	1761.8203	0.0921	0	47	0.0032	1	R.EILSVDCSTNNPSQAK.L
☑	412	937.6735	1873.3324	1872.9833	0.3491	0	17	2.5	1	K.LFDSDPITVTVPVEVSR.K

Fixed modifications: Carbamidomethyl (C)
Variable modifications: Oxidation (M)
Cleavage by Trypsin: cuts C-term side of KR unless next residue is P
Sequence Coverage: **15%**

Matched peptides shown in **Bold Red**

1 MMKTLLLFVG LLLTWESGQV LGDQTVSDNE LQEMSNQGSK YVNKEIQNAV
51 NGVKQIKTLI EKTNEERK**TL** **LSNLEEAK**KK KEDALNETRE SETKLKELPG
101 VCNETMMALW EECKPCLKQT CMKFYARVCR SGSGLVGRQL EEFLNQSSPF
151 YFWMNGDRID SLLENDRQQT HMLDVMQDHF SRASSIIDEL FQDRFFTREP
201 QDTYHYLPFS LPHRRPHFFF PKSRIVRSLM PFSPYEPLNF HAMFQPFLEM
251 IHEAQQAMDI HFHSPAFQHP PTEFIREGDD DRTVCREIRH NSTGCLRMKD
301 QCDKCR**EILS** **VDCSTNNPSQ** **AK**LRR**ELDES** **LQVAER**LTRK YNELLKSYQW
351 KMLNTSSLLE QLNEQFNWVS RLANLTQGED QYYLRVTTVA SHTSDSDVPS
401 GVTEVVVK**LF** **DSDPITVTVP** **VEVSR**KNPKF **METVAEKALQ** **EYR**KKHREE

Start - End	Observed	Mr(expt)	Mr(calc)	Delta	Miss	Sequence
69 - 78	559.3435	1116.6724	1116.6026	0.0698	0	K.TLLSNLEEAK.K (Ions score 36)
307 - 322	881.9635	1761.9124	1761.8203	0.0921	0	R.EILSVDCSTNNPSQAK.L (Ions score 47)
326 - 336	644.8985	1287.7824	1287.6306	0.1518	0	R.ELDESLQVAER.L (Ions score 28)
409 - 425	937.6735	1873.3324	1872.9833	0.3491	0	K.LFDSDPITVTVPVEVSR.K (Ions score 17)
430 - 437	970.3040	969.2967	969.4477	-0.1510	0	K.FMETVAEK.A Oxidation (M) (Ions score 24)
438 - 443	779.2860	778.2787	778.3973	-0.1186	0	K.ALQEYR.K (Ions score 17)

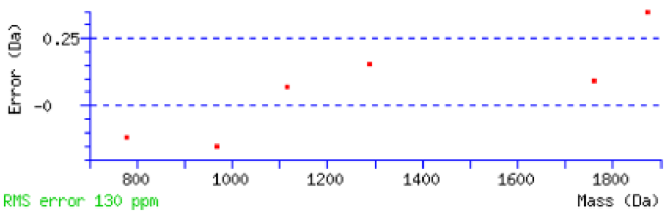

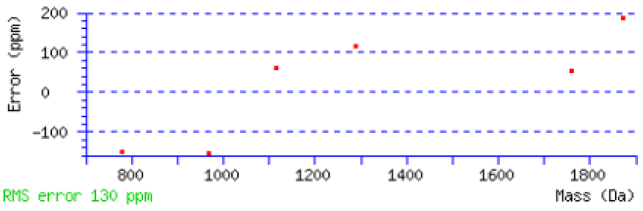

(D) Mascot Search Result HT2482 (RET4=RBP4)

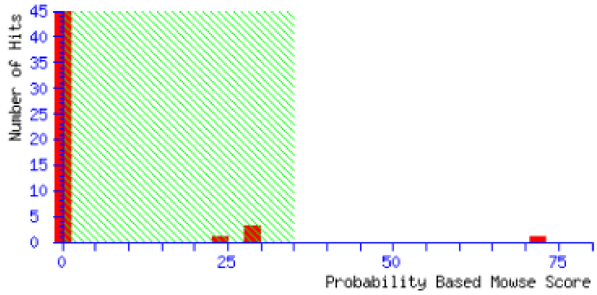

1. sp|P02753|RET4_HUMAN **Mass:** 23337 **Score:** 72 **Queries matched:** 10 **emPAI:** 0.83
Retinol-binding protein 4 OS=Homo sapiens GN=RBP4 PE=1 SV=3
Check to include this hit in error tolerant search or archive report

Query	Observed	Mr(expt)	Mr(calc)	Delta	Miss	Score	Expect	Rank	Peptide
95	879.4150	878.4077	878.4498	-0.0420	1	22	1.5	1	R.VKENFDK.A
119	509.8415	1017.6684	1017.4913	0.1771	0	46	0.0064	1	R.QEELCLAR.Q
148	583.1120	1164.2094	1164.5775	-0.3680	0	(12)	13	4	R.DPNGLPPEAQK.I
150	583.8600	1165.7054	1164.5775	1.1280	0	46	0.0047	1	R.DPNGLPPEAQK.I
152	589.3580	1176.7014	1176.5274	0.1741	0	24	0.94	1	R.FSGTWYAMAK.K + Oxidation (M)
155	599.9900	1197.9654	1197.6182	0.3472	0	38	0.031	1	K.YWGVASFLQK.G
170	1303.5970	1302.5897	1302.6139	-0.0242	0	(15)	5.6	2	R.LIVHNGYCDGR.S
172	652.4570	1302.8994	1302.6139	0.2855	0	(32)	0.13	1	R.LIVHNGYCDGR.S
173	652.6045	1303.1944	1302.6139	0.5805	0	40	0.019	1	R.LIVHNGYCDGR.S
175	1305.4810	1304.4737	1302.6139	1.8598	0	(27)	0.34	1	R.LIVHNGYCDGR.S

Fixed modifications: Carbamidomethyl (C)
Variable modifications: Oxidation (M)
Cleavage by Trypsin: cuts C-term side of KR unless next residue is P
Sequence Coverage: **28%**

Matched peptides shown in **Bold Red**

1 MKWVWALLLL AALGSGRAER DCRVSSFR**VK** **ENFDK**AR**FSG** **TWYAMAK**KDP
51 EGLFLQDNIV AEFSVDETGQ MSATAKGRVR LLNNWDVCAD MVGTFTDTED
101 PAKFKMK**YWG** **VASFLQK**GND DHWIVDTDYD TYAVQYSCRL LNLDGTCADS
151 YSFVFSR**DPN** **GLPPEAQK**IV RQR**QEELCLA** **R**QYR**LIVHNG** **YCDGR**SERNL
201 L

Start - End	Observed	Mr(expt)	Mr(calc)	Delta	Miss	Sequence
29 - 35	879.4150	878.4077	878.4498	-0.0420	1	R.VKENFDK.A (Ions score 22)
38 - 47	589.3580	1176.7014	1176.5274	0.1741	0	R.FSGTWYAMAK.K Oxidation (M) (Ions score 24)
108 - 117	599.9900	1197.9654	1197.6182	0.3472	0	K.YWGVASFLQK.G (Ions score 38)
158 - 168	583.1120	1164.2094	1164.5775	-0.3680	0	R.DPNGLPPEAQK.I (Ions score 12)
158 - 168	583.8600	1165.7054	1164.5775	1.1280	0	R.DPNGLPPEAQK.I (Ions score 46)
174 - 181	509.8415	1017.6684	1017.4913	0.1771	0	R.QEELCLAR.Q (Ions score 46)
185 - 195	1303.5970	1302.5897	1302.6139	-0.0242	0	R.LIVHNGYCDGR.S (Ions score 15)
185 - 195	652.4570	1302.8994	1302.6139	0.2855	0	R.LIVHNGYCDGR.S (Ions score 32)
185 - 195	652.6045	1303.1944	1302.6139	0.5805	0	R.LIVHNGYCDGR.S (Ions score 40)
185 - 195	1305.4810	1304.4737	1302.6139	1.8598	0	R.LIVHNGYCDGR.S (Ions score 27)

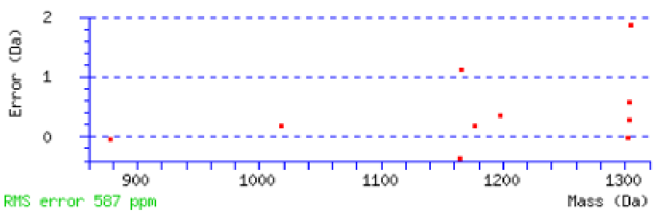

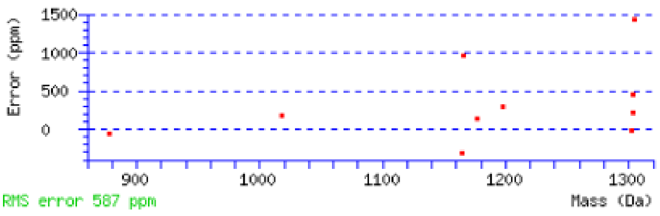

(E) Mascot Search Result HT1248 (fetuin-A)

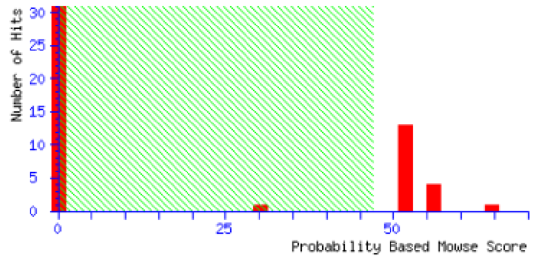

1. sp|P02765|FETUA_HUMAN **Mass:** 40098 **Score:** 65 **Queries matched:** 3 **emPAI:** 0.20
Alpha-2-HS-glycoprotein OS=Homo sapiens GN=AHSG PE=1 SV=1
☐ Check to include this hit in error tolerant search or archive report

	Query	Observed	Mr(expt)	Mr(calc)	Delta	Miss	Score	Expect	Rank	Peptide
	137	847.2330	846.2257	846.4269	-0.2012	0	32	2.7	4	K.CNLLAEK.Q
☑	242	598.8240	1195.6334	1195.6197	0.0138	0	56	0.0076	1	**K.HTLNQIDEVK.V**
☑	544	700.0970	2097.2692	2096.0110	1.2582	0	52	0.013	1	**R.HTFMGVVSLGSPSGEVSHPR.K + Oxidatior**

2. sp|P17412|FRDA_WOLSU **Mass:** 73300 **Score:** 56 **Queries matched:** 1 **emPAI:** 0.05
Fumarate reductase flavoprotein subunit OS=Wolinella succinogenes GN=frdA PE=1 SV=3
☐ Check to include this hit in error tolerant search or archive report

	Query	Observed	Mr(expt)	Mr(calc)	Delta	Miss	Score	Expect	Rank	Peptide
☑	146	452.8865	903.7584	903.4848	0.2737	0	56	0.011	1	**R.MAIINAQK.T + Oxidation (M)**

Fixed modifications: Carbamidomethyl (C)
Variable modifications: Oxidation (M)
Cleavage by Trypsin: cuts C-term side of KR unless next residue is P
Sequence Coverage: **10%**

Matched peptides shown in **Bold Red**

1 MKSLVLLLCL AQLWGCHSAP HGPGLIYRQP NCDDPETEEA ALVAIDYINQ
51 NLPWGYK**HTL NQIDEVK**VWP QQPSGELFEI EIDTLETTCH VLDPTPVARC
101 SVRQLKEHAV EGDCDFQLLK LDGKFSVVYA KCDSSPDSAE DVRKVCQDCP
151 LLAPLNDTRV VHAAKAALAA FNAQNNGSNF QLEEISRAQL VPLPPSTYVE
201 FTVSGTDCVA KEATEAAK**CN LLAEK**QYGFC KATLSEKLGG AEVAVTCTVF
251 QTQPVTSQPQ PEGANEAVPT PVVDPDAPPS PPLGAPGLPP AGSPPDSHVL
301 LAAPPGHQLH RAHYDLR**HTF MGVVSLGSPS GEVSHPR**KTR TVVQPSVGAA
351 AGPVVPPCPG RIRHFKV

Start - End	Observed	Mr(expt)	Mr(calc)	Delta	Miss	Sequence
58 - 67	598.8240	1195.6334	1195.6197	0.0138	0	**K.HTLNQIDEVK.V** (Ions score 56)
219 - 225	847.2330	846.2257	846.4269	-0.2012	0	**K.CNLLAEK.Q** (Ions score 32)
318 - 337	700.0970	2097.2692	2096.0110	1.2582	0	**R.HTFMGVVSLGSPSGEVSHPR.K** Oxidation (M) (Ions s

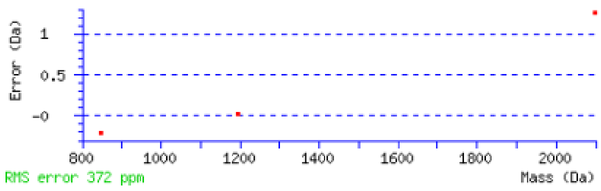

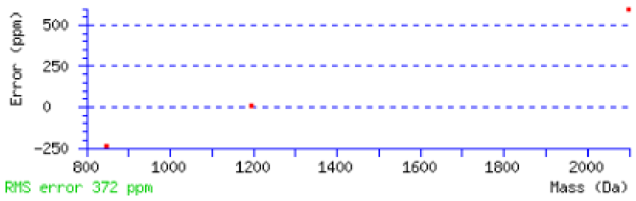

(F) Mascot Search Result HT1240 (VDBP)

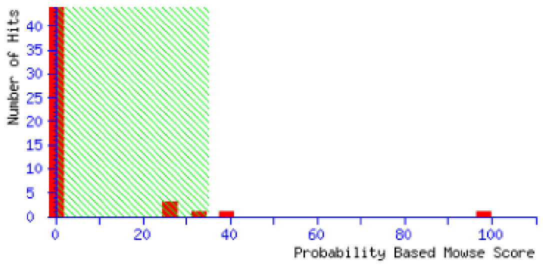

1. sp|P02774|VTDB_HUMAN **Mass:** 54526 **Score:** 98 **Queries matched:** 16 **emPAI:** 0.30
Vitamin D-binding protein OS=Homo sapiens GN=GC PE=1 SV=1
Check to include this hit in error tolerant search or archive report

Query	Observed	Mr(expt)	Mr(calc)	Delta	Miss	Score	Expect	Rank	Peptide
13	527.2930	526.2857	526.3479	-0.0621	0	15	3.9	1	K.GPLLK.K
70	799.2470	798.2397	798.4851	-0.2454	0	25	0.72	1	K.VLEPTLK.S
71	817.3360	816.3287	816.4130	-0.0843	0	19	3.6	7	K.EFSHLGK.E
83	458.2465	914.4784	914.4498	0.0287	0	35	0.076	1	K.YTFELSR.R
84	938.3020	937.2947	937.4756	-0.1809	0	25	0.66	2	K.ELSSFIDK.G
86	938.4110	937.4037	937.4756	-0.0719	0	(20)	1.9	3	K.ELSSFIDK.G
88	950.4010	949.3937	949.4539	-0.0601	0	10	22	3	K.LCDNLSTK.N
89	952.4160	951.4087	951.5025	-0.0938	0	(17)	4	2	K.ELPEHTVK.L
90	476.7950	951.5754	951.5025	0.0729	0	28	0.3	1	K.ELPEHTVK.L
98	503.1695	1004.3244	1004.4233	-0.0989	0	24	1	1	K.DVCDPGNTK.V
118	558.2930	1114.5714	1114.4059	0.1655	0	40	0.021	1	K.FEDCCQEK.T
128	585.9205	1169.8264	1169.6445	0.1820	0	31	0.16	1	R.THLPEVFLSK.V
147	638.3255	1274.6364	1274.5601	0.0763	0	27	0.44	2	R.VCSQYAAYGEK.K
210	732.2500	1462.4854	1462.4833	0.0022	0	40	0.016	1	K.CCESASEDCMAK.E + Oxidation (M)
283	784.3155	1566.6164	1565.8090	0.8075	0	74	6.6e-006	1	K.FPSGTFEQVSQLVK.E
339	566.2310	1695.6712	1693.9039	1.7672	1	13	7.6	8	R.KFPSGTFEQVSQLVK.E

2. sp|Q9NZU1|FLRT1_HUMAN **Mass:** 72055 **Score:** 36 **Queries matched:** 1 **emPAI:** 0.05
Leucine-rich repeat transmembrane protein FLRT1 OS=Homo sapiens GN=FLRT1 PE=1 SV=3
Check to include this hit in error tolerant search or archive report

Query	Observed	Mr(expt)	Mr(calc)	Delta	Miss	Score	Expect	Rank	Peptide
480	920.5823	2758.7252	2760.3454	-1.6202	1	36	0.019	1	K.LHLDDNSVSTVSIEEDAFADSKQLK.L

Fixed modifications: Carbamidomethyl (C)
Variable modifications: Oxidation (M)
Cleavage by Trypsin: cuts C-term side of KR unless next residue is P
Sequence Coverage: **24%**

Matched peptides shown in **Bold Red**

1 MKRVLVLLLA VAFGHALERG RDYEKNKVCK **EFSHLGK**EDF TSLSLVLYSR
51 **KFPSGTFEQV SQLVK**EVVSL TEACCAEGAD PDCYDTRTSA LSAKSCESNS
101 PFPVHPGTAE CCTKEGLERK LCMAALKHQP QEFPTYVEPT NDEICEAFRK
151 DPKEYANQFM WEYSTNYGQA PLSLLVSYTK SYLSMVGSCC TSASPTVCFL
201 KERLQLKHLS LLTTLSNR**VC SQYAAYGEK**K SRLSNLIKLA QKVPTADLED
251 VLPLAEDITN ILSK**CCESAS EDCMAKELPE HTVKLCDNLS TK**NSK**FEDCC**
301 **QEK**TAMDVFV CTYFMPAAQL PELPDVELPT NK**DVCDPGNT K**VMDK**YTFEL**
351 **SR**R**THLPEVF LSKVLEPTLK** SLGECCDVED STTCFNAK**GP LLK**K**ELSSFI**
401 **DK**GQELCADY SENTFTEYKK KLAERLKAKL PDATPKELAK LVNKRSDFAS
451 NCCSINSPPL YCDSEIDAEL KNIL

Start - End	Observed	Mr(expt)	Mr(calc)	Delta	Miss	Sequence
31 - 37	817.3360	816.3287	816.4130	-0.0843	0	K.EFSHLGK.E (Ions score 19)
51 - 65	566.2310	1695.6712	1693.9039	1.7672	1	R.KFPSGTFEQVSQLVK.E (Ions score 13)
52 - 65	784.3155	1566.6164	1565.8090	0.8075	0	K.FPSGTFEQVSQLVK.E (Ions score 74)
219 - 229	638.3255	1274.6364	1274.5601	0.0763	0	R.VCSQYAAYGEK.K (Ions score 27)
265 - 276	732.2500	1462.4854	1462.4833	0.0022	0	K.CCESASEDCMAK.E Oxidation (M) (Ions score 40)
277 - 284	952.4160	951.4087	951.5025	-0.0938	0	K.ELPEHTVK.L (Ions score 17)
277 - 284	476.7950	951.5754	951.5025	0.0729	0	K.ELPEHTVK.L (Ions score 28)
285 - 292	950.4010	949.3937	949.4539	-0.0601	0	K.LCDNLSTK.N (Ions score 10)
296 - 303	558.2930	1114.5714	1114.4059	0.1655	0	K.FEDCCQEK.T (Ions score 40)
333 - 341	503.1695	1004.3244	1004.4233	-0.0989	0	K.DVCDPGNTK.V (Ions score 24)
346 - 352	458.2465	914.4784	914.4498	0.0287	0	K.YTFELSR.R (Ions score 35)
354 - 363	585.9205	1169.8264	1169.6445	0.1820	0	R.THLPEVFLSK.V (Ions score 31)
364 - 370	799.2470	798.2397	798.4851	-0.2454	0	K.VLEPTLK.S (Ions score 25)
389 - 393	527.2930	526.2857	526.3479	-0.0621	0	K.GPLLK.K (Ions score 15)
395 - 402	938.3020	937.2947	937.4756	-0.1809	0	K.ELSSFIDK.G (Ions score 25)
395 - 402	938.4110	937.4037	937.4756	-0.0719	0	K.ELSSFIDK.G (Ions score 20)

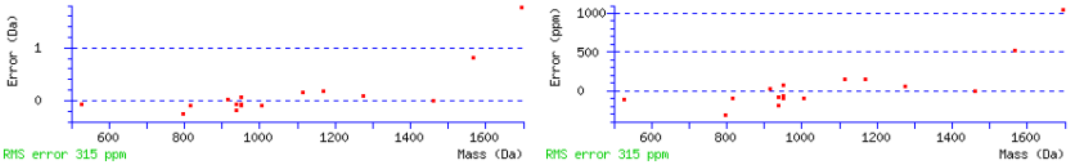

Supplement: Additional file 1 — Figure S1 - Mascot search results--Information about the identified proteins obtained using the Mascot server. (A) Mascot search result for T2116 (clusterin) (B) Mascot Search Result for T2103 (clusterin) EST (C) Mascot search result for T1486 (clusterin) (D) Mascot search result for HT2482 (RET4 = RBP4) (E) Mascot search result for HT1248 (fetuin-A) (F) Mascot search result for HT1240 (VDBP). [file 1471-2334-11-71-S1.PDF]
